# Confusion, cognitive impairment, and spinal cord compression caused by plasmacytoma: a case report

**DOI:** 10.1186/s12883-021-02332-3

**Published:** 2021-08-06

**Authors:** Michael Axenhus, Nenad Bogdanovic

**Affiliations:** 1 Department of Neurobiology, Care Sciences and Society, Section of Neurogeriatrics, Center for Alzheimer Research, Karolinska Institute, Solna, Sweden; 2 Department of Neurobiology, Care Sciences and Society, Division of Clinical Geriatrics, Karolinska Institute, Solna, Sweden; 3 Theme Inflammation and Aging, Karolinska University Hospital, Huddinge, Sweden

**Keywords:** Case report, Cognitive impairment, Dementia, Plasmacytoma

## Abstract

**Background:**

Plasmacytomas are rare tumors comprised of neoplastic monoclonal plasma cells and can be found anywhere in the body. Plasmacytomas that involve the nervous system can give rise to diffuse symptoms depending on their location. Patients with confusion or dementia might be difficult to neurologically assess in an acute setting and the subtle symptoms of neurological pathology caused by rare malignancies might go undiagnosed.

**Case presentation:**

The patient is an 80 year old man presenting to the ER with walking difficulties, pain, and confusion. He underwent neurological evaluation for dementia and was eventually diagnosed with possible Alzheimer’s disease and a malignant plasmacytoma causing spinal cord compression. His CSF sample showed normal amyloid rate and very low Aβ. Following rehabilitation and oncological treatment, his walking ability and confusion improved.

**Conclusion:**

This case is unique as we demonstrate that spinal cord compression by plasmacytoma can lead to abnormal CSF levels of several known pathology markers for Alzheimer’s disease and neuronal damage. We suggest that highly divergent amyloid CSF levels could be indicative of spinal pathologies affecting CSF circulation. We also suggest closer assessment of elderly confusion patients in ER settings by consultants specialized in neurological disorders.

## Background

Elderly patients with dementia or confusion are a common sight in emergency rooms (ER) and hospitals around the world [1, 2]. Acute confusion, particularly in the elderly, have a broad range of causes and can develop as a result of infections, fractures or strokes [3]. Plasmacytoma is a rare tumor and can be found in any tissue. We show, to our knowledge, the first case of spinal cord compression caused by plasmacytoma presenting with confusion as a primary symptom. We also show abnormal cerebrospinal fluid (CSF) pathology marker levels caused by spinal cord compression.

We report the case of an 80 year old man that came to the ER with hip pain and confusion. He was treated as a mild trauma patient and diagnosed with hip arthritis. His confusion was judged to be caused by pain. He was admitted to a specialized neurogeriatric ward, which at the time worked as a general geriatric ward due to constraints brought on by Covid-19. Further examination and tests revealed extensive neurological symptoms and the patient was later diagnosed with both possible Alzheimer’s disease (AD) and a spinal cord compressing metastasized plasmacytoma resulting in highly divergent amyloid CSF levels.

This report demonstrate that dementia and confusion patients are clinically difficult groups of patients that require careful assessment and handling when in an ER setting. Neurological symptoms in these patient groups might be missed and ER clinicians should keep close contact with physicians that have experience of these patient groups, such as neurological or geriatric specialties, in order to evaluate the possibility of newly developed underlying pathology.

## Case presentation

The patient was an 80 year old man admitted to the ER with abdominal pain. At admittance, the patient was unable to walk and described an increased difficulty with movement and coordination of the lower limbs, tendency to fall, extensive fatigue, and pain. Six months earlier, the patient had full movement of his lower limbs and could walk unaided. He described an increased intensity and duration of symptoms during the last three months. He was also confused and was unable to answer correctly on orienting questions. Blood serum samples taken at the ER showed a slight thrombocytopenia and mild kidney insufficiency. An arterial blood gas showed hypercalcemia. His condition was deemed to be a combination of somatic pain and confusion. He was therefore treated as a mild trauma patient. Computer tomography (CT) of the abdomen was performed and a surgical consult ruled out any surgical pathology. A pelvis CT showed deterioration of the right hip and an orthopedic consult diagnosed right-sided hip arthritis. The patient was admitted to the orthopedic trauma ward for one day before being transferred to the geriatric clinic for mobilization and pain optimization. He was placed at the neurogeriatric ward to due to space constraints caused by Covid-19.

At the neurogeriatric ward, clinical examination revealed several neurological abnormalities including apraxia, dysdiadochokinesia, intentional tremor, clonus, and parkinsonism. Both sides of his body were affected although the left side showed more pronounced symptoms than the right. He was paraspastic and there was parkinsonism in his upper extremities with cogwheel rigidity bilaterally. There was hyporeactivity in his triceps, biceps and brachioradialis tendon reflexes. His lower body display mixed neurological symptoms with peripheral rigidity and hyporeactivity in quadriceps tendons reflexes. He admitted no pain sensation in his left lower leg and foot, though both proprioception and sense of vibration were preserved. He had positive Babinskis sign bilaterally, indicative of damage to the pyramidal neuronal pathways as well as Glabellar tap sign indicating a primitive frontal release, concurrent with parkinsonism. He had difficulty with coordination and was unable to perform fine motor skill tests using his left hand. He was only capable of a wide gait and unable to performed quick turns or adjust walking speed even while using walking aid. He had orthostatic hypotension and was prone to falling. His Mini Mental State Examination score was 9/30 and he had problems with short-term memory, orientation and recall (Table 1).

**Table 1:** Mini Mental State Exam score showing advanced signs of cognitive impairment and deficits in orientation, recall, and attention

MMSE Category	Patient score	Max score
Orientation	2	10
Recall, Immediate	1	3
Attention and Calculation	1	5
Recall, Delayed	1	3
Language	4	8
Copying	0	1
Total score	9	30

Anamnesis from the patient and his next of kin revealed that he had been suffering from symptoms such as diarrhea, urine retention, fatigue, and pain for at least six months. The symptoms were infrequent but had become more and more common before admittance to the hospital. He had also lost weight, estimated at 20% of his body weight. His partner noted that he had become more forgetful and occasionally did not recognize acquaintances and family members. He had trouble walking and moving around the house. Sometimes he would appear to be confused about everyday tasks. There was no family history of dementia or cognitive impairment. The patient had worked as a salesperson up until retirement at age 67 and had up until six months before admittance to the hospital not shown any signs of memory impairment or dementia. His deterioration, by all accounts, had been sudden and quick. He was subsequently treated as a patient with suspected cognitive impairment of an unknown cause and standard examinations and tests followed.

**Fig. 1:**
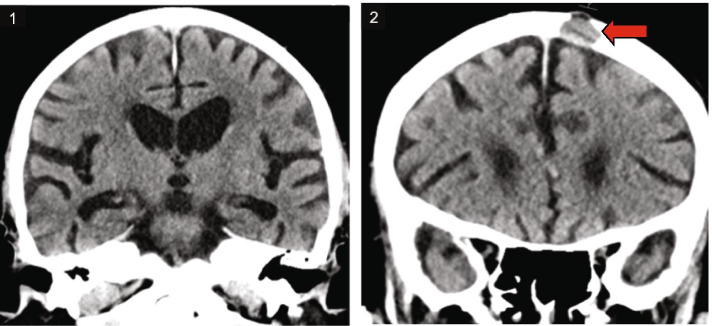
Brain CT images showing white matter changes Fazekas 2, cortical atrophy GCA1-2, medial temporal lobe atrophy with reduced hippocampus, MTL 3 (1) as well as a single lytic metastasis within the left side of the frontal bone (red arrow) (2)

**Fig. 2:**
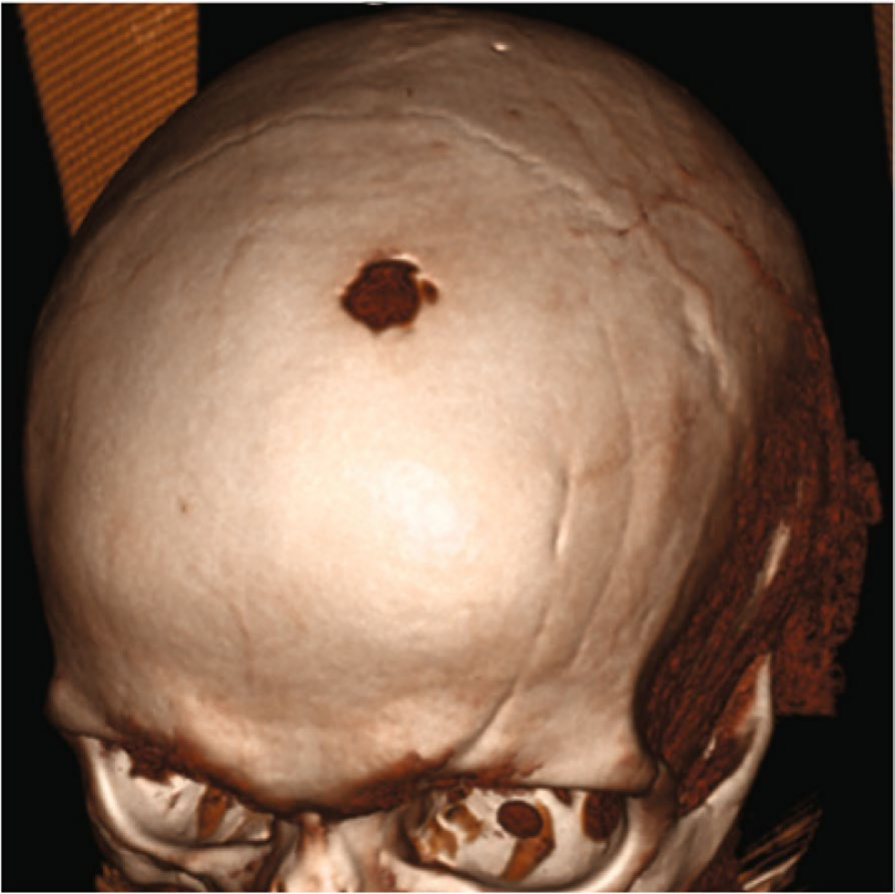
3D reconstruction of cranial bone structure showing total lysis of the frontal bone with intrusion into the brain cavity

A brain CT revealed white matter changes corresponding to Fazekas stage 2 and medial temporal atrophy grade 2–3. The hippocampus was significantly reduced in volume bilaterally (Fig. 1). There was a light global atrophy and no signs of increased intracranial pressure. There was a lytic destruction in the left frontal bone, indicative of a metastasis (Fig. 2).

ApoE analysis showed E3/E3 variant. Electroencephalography that showed marked abnormality with laterality to the left hemisphere and loss of alpha rhythm. Extended blood panel showed highly elevated serum calcium and lactate dehydrogenase.

CSF analysis showed increased albumin at 1230 mg/L (ref < 400 mg/L), severely reduced Amyloid beta (Aβ) 42 at 265 ng/L but with normal amyloid ratio at 0,9 (ref > 0,59), elevated neurofilament light protein at 4600 ng/L. Tau protein as well as phosphorylated Tau protein were within normal range values. Immunoglobulin assay showed increased levels. Paraneoplastic antibodies were negative.

A diagnosis of possible AD was confirmed using findings of degenerative changes on brain CT, clinical status, and pathological electroencephalography. However, CSF amyloid biomarkers findings did not correlate to AD. Furthermore, neither the full spectrum of his neurological symptoms nor the very high CSF albumin levels could be explained by an AD diagnosis. These findings, coupled with the lytic destructive lesion in the frontal bone and pronounced paraspasticity, prompted further examination.

**Fig. 3:**
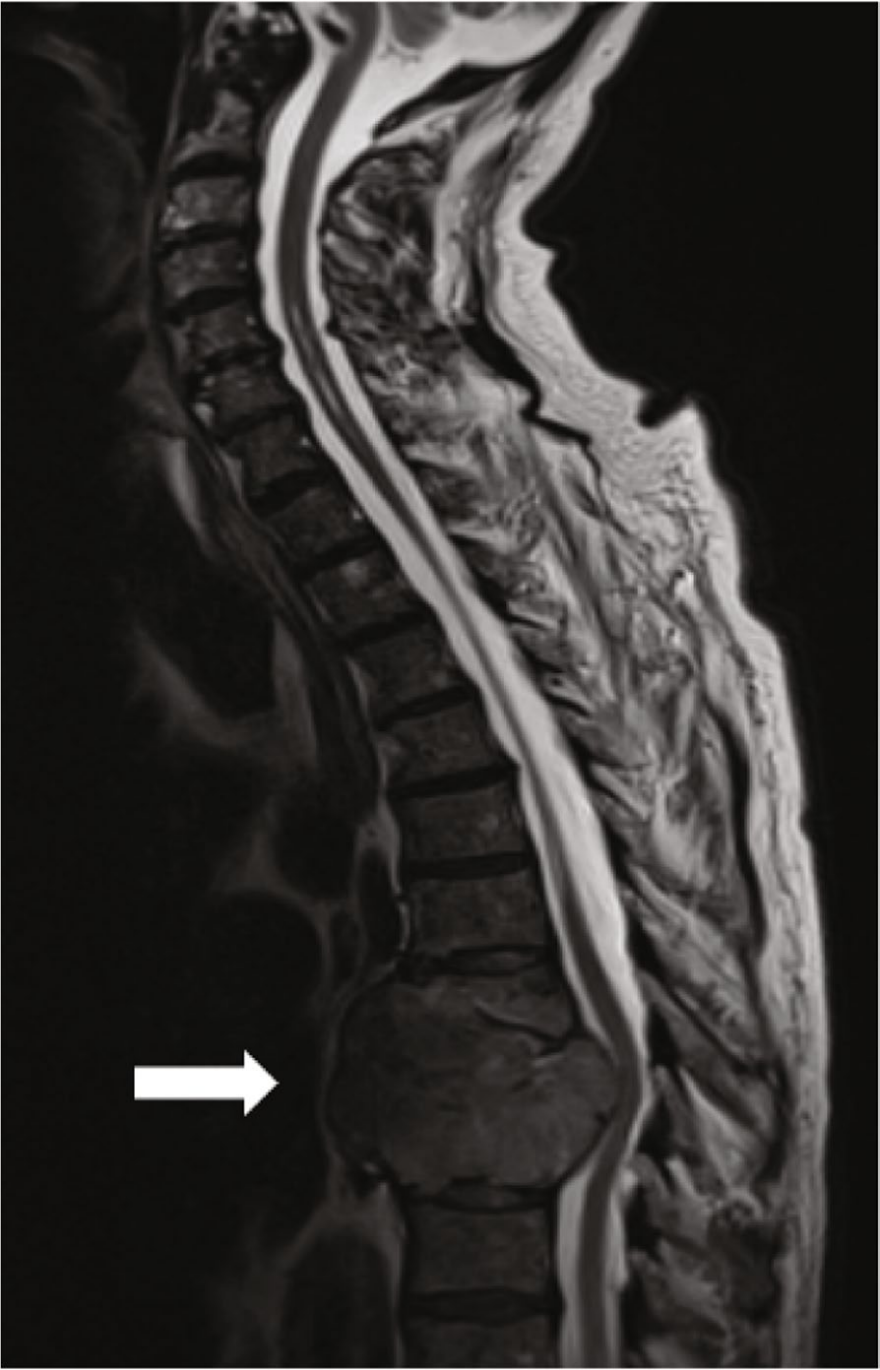
Spinal magnetic resonance image show plasmacytoma (white arrow) encapsulating the whole of Th8 with severe spinal cord compression with subsequent myelopathy

A spinal MRI revealed a large lytic tumor incapsulating the whole of Th8 as well as parts of Th7 and Th9 (Fig. 3). Metastasis were found throughout the spine and the pelvis. Biopsy and immunocytochemistry confirmed the tumor to be a plasmacytoma containing both necrotic tissue and abnormal plasma cells. The primary tumor compressed the medulla spinalis and myelopathy was present within the spinal cord in association with the tumor.

### Treatment

The patient received oncological and hematological consultations and was then started on high dose Prednisolone, Bortezomib and radiation therapy. Neurosurgical intervention was dismissed considering the large involvement of the tumor and the already significant tissue damage. The patient was kept at a geriatric emergency ward and received both medical treatment as well as daily rehabilitation and physical therapy.

### Outcome

Treatment enabled some reduction in tumor size and after one month the patient experienced slight improvement with absence of tremor and improved walking ability. His rigidity was also less substantial, and he experienced less fatigue. Although his cognitive abilities were not improved, neither had they deteriorated any further. His prognosis at this stage was considered good given his response to treatment.

## Discussion and conclusion

The patient reported here initially presented to the ER with abdominal pain and confusion. The patient had a six-month history of rapid onset cognitive impairment and newly developed neurological symptoms. The confusion was judged to be brought on by pain and the patient was admitted for a newly diagnosed hip arthritis. Since neurological examination was not performed at the time of admission the underlying pathologies were not detected.

The patient was later revealed to suffer from neurological symptoms indicative of cognitive decline and neurological pathology. Possible AD as well as a metastasized plasmacytoma were diagnosed. There were signs of partial spinal cord compression as he did not experience pain on his lower left side but still had a sense proprioception and vibration. Pyramidal symptoms were also present with bilateral positive Babinski sign and spasticity. These symptoms, as well as the patient’s rapid development of cognitive impairment would have been apparent already at the time of admission to the ER and indicative of routine neurological and radiological examinations.

Cases of neurological deficit brought on by spinal plasmacytoma are rare and the literature is limited to case reports. Plasmacytoma can develop anywhere along the axial skeleton, although most commonly within the thoracic spine. There are no typical clinical findings associated with spinal plasmacytoma but pathological mechanisms including destruction of supporting bone structure and compression of the spinal cord produce symptoms such as pain, paraparesis, and paraplegia. Although rarer symptoms can also occur (Table 2).

**Table 2:** Case reports of neurological symptoms caused by spinal plasmacytoma

Author	Year	Patients	Age/Sex	Neurological symtoms
Baba H. et al. [4]	1998	8 (2 described)	58/woman 73/man	back pain and hypoesthesia L4/L5 weakness of arms and sensory deficit below C5
Takahashi T. et al.[5]	1998	2	72/man 77/man	progressive paraparesis in both cases
Ho J. et al.[6]	2017	1	69/man	lower right radiculopathy
Afonso PD. et al.[7]	2010	1	41/man	back pain, paraparesis, and hyporeflexia of the lower limbs
Fridman A. et al.[8]	2009	1	46/woman	Horner’s syndrome
Finsterer J*. *et al. [9]	2002	1	47/man	transverse syndrome with pain and sensory disturbance
Terada T. et al.[10]	2011	1	53/woman	paraplegia and back pain
Eseonu KC. et al.[11]	2020	1	71/woman	paraplegia

No case with confusion as a primary symptom has been reported. Our case is the first case that include analysis of CSF levels of common AD markers in a patient with spinal cord compressing plasmacytoma.

There was evidence of axonal damage in the form of elevated CSF levels of neurofilament light protein, caused by the compression of the medulla spinalis and indirect effect on the white matter of the brain. CSF levels of Aβ were extremely low but not amyloid rate and CT brain showed a neurodegenerative finding concurrent with AD [12]. Interestingly, the patients ApoE variant was E3/E3 and Tau CSF levels were normal. The very low Aβ42 and the normal amyloid rate could therefore not solely be explained by the patient’s AD. It is likely that decreased Aβ in the CSF and high albumin level occurred as a result of reduced CSF circulation under the level of the medulla spinalis compression. Normal amyloid rate with low Aβ42 and Aβ40 levels is a CSF constitution which is not characteristic for AD [13]. The relationship between spinal cord compression and AD CSF markers is under debate but studies into CSF circulation indicate that decreased CSF circulation results in increased amyloid levels [14–16]. Spinal pathologies that affect CSF circulation could therefore be assumed to change amyloid levels as well. Disruption in CSF circulation are also often evidenced by immunoglobulin testing, albumin increase, and the elevation in neurofilament light chain protein levels, similar to the findings in this case [17, 18].

Since the lumbar puncture was taken from the region below the compression, the biomarker dynamics in the CSF over the compression cannot be determined. It is possible that CSF sampling from above the medulla spinalis compression may reflect typical AD biomarker pattern. Since the patient had both proprioception and sense of vibration in the lower half of his body, a total stoppage of the medulla spinalis is unlikely. However, it was clear that some disturbance in CSF circulation was occurring.

Because of its rarity and multitude of manifestations, plasmacytoma can sometimes be mistaken for other spinal lesions such as metastasis, gliomas, or other hematological malignancies [19–21]. First line treatment for plasmacytoma is radiotherapy, corticosteroids, chemotherapy, and/or immunotherapy rather than surgical resection [22]. Cases in the literature have been reported wherein misdiagnosed plasmacytomas have been surgically resected [10, 21, 23]. Accurate diagnostics is therefore essential in the treatment of plasmacytomas. Diffuse neurological symptoms can be a challenge to properly assess, particularly in confused and elderly patients.

In this case, the patient’s neurological symptoms were an effect of both his dementia and the spinal cord compression caused by his malignancy. The diagnosis set at the ER is important as it influence the rest of the patient stay at the hospital, something which was demonstrated in this case as proper care was delayed because of the initial assessment at the ER. The clinical status and anamnesis of dementia and confusion patients are often complicated and difficult to clinically assess without experience. This represents an increased risk factor for an already vulnerable patient category and a challenge for ER physicians. We recommend that elderly patients that present with acute confusion should be evaluated for neurological symptoms. In patients with suspected or confirmed dementia, consultations with specialists who commonly deal with dementia and confusion, such as psychiatric, neurological, or geriatric specialists, would be indicated.

Sampling of the CSF displayed remarkable divergent amyloid levels caused by spinal cord compression. We therefore suggest that patients with severely abnormal CSF protein levels, in particular normal amyloid rate with simultaneous low Aβ42, if no other reasonable clinical explanation can be found, should be evaluated for pathologies affecting CSF circulation.

In conclusion, this case demonstrates that the diagnosis and treatment of neurological symptoms in geriatric patients, particularly those with potential neurocognitive disorders and confusion, represent a difficult challenge for ER physicians. We also suggest that severely abnormal amyloid CSF levels, without apparent cause, could be indicative of pathologies affecting CSF circulation.

## Data Availability

Because of privacy concerns, we are unable to provide any additional clinical data due to the unique characteristics of this case.

## Abbreviations

AD: Alzheimer’s disease
Aβ: Amyloid β
CSF: Cerebrospinal fluid
ER: Emergency Room
